# Genetic variability in Brazilian wheat cultivars assessed by microsatellite markers

**DOI:** 10.1590/S1415-47572009005000045

**Published:** 2009-09-01

**Authors:** Ivan Schuster, Elisa Serra Negra Vieira, Glacy Jaqueline da Silva, Francisco de Assis Franco, Volmir Sérgio Marchioro

**Affiliations:** 1 Cooperativa Central de Pesquisa Agrícola, Cascavel, PRBrazil; 2 Departamento de Ciências Biológicas, Universidade Paranaense, Cascavel, PRBrazil

**Keywords:** *Triticum aestivum*, germplasm, cultivar characterization, cluster analysis, molecular markers

## Abstract

Wheat (*Triticum aestivum*) is one of the most important food staples in the south of Brazil. Understanding genetic variability among the assortment of Brazilian wheat is important for breeding. The aim of this work was to molecularly characterize the thirty-six wheat cultivars recommended for various regions of Brazil, and to assess mutual genetic distances, through the use of microsatellite markers. Twenty three polymorphic microsatellite markers (PMM) delineated all 36 of the samples, revealing a total of 74 simple sequence repeat (SSR) alleles, *i.e.* an average of 3.2 alleles per locus. Polymorphic information content (PIC value) calculated to assess the informativeness of each marker ranged from 0.20 to 0.79, with a mean of 0.49. Genetic distances among the 36 cultivars ranged from 0.10 (between cultivars Ocepar 18 and BRS 207) to 0.88 (between cultivars CD 101 and Fudancep 46), the mean distance being 0.48. Twelve groups were obtained by using the unweighted pair-group method with arithmetic means analysis (UPGMA), and thirteen through the Tocher method. Both methods produced similar clusters, with one to thirteen cultivars per group. The results indicate that these tools may be used to protect intellectual property and for breeding and selection programs.

Although Brazil is not as yet self-sufficient in wheat (*Triticum aestivum*) production and depends on importation to supply its domestic requirements, wheat has already become an important crop, especially in the south, in the states of Paraná and Rio Grande do Sul. As a winter crop, it is an income-option for farmers who grow soybean or corn in the summer. In 2007, Brazil produced 3.82 million tons of wheat on 1.82 million ha of land, 90% of which was in Rio Grande do Sul and Paraná (Conab, 2008).

Although several types of molecular markers have been employed to evaluate genetic diversity in wheat, such as random amplified polymorphic DNA (RAPD) (Joshi and Nguyen, 1993), restriction fragment-length polymorphism (RFLP) (Siedler *et al.*, 1994, Kim and Ward, 2000), amplified fragment-length polymorphism (AFLP) (Barret and Kidwell, 1998, Burkhamer *et al.*, 1998), sequence tagged-site (STS) (Chen *et al.*, 1994) and inter-simple sequence repeat (ISSR) (Devos and Galley, 1992; Nagaoka and Ogihara, 1997), microsatellite markers have been suggested as the most informative method for this type of analysis (Röder *et al.*, 1998a, 1998b). Since they are multiallelic, chromosome-specific and well distributed in the genome, microsatellite markers have already been used with wheat for selecting specific genes (Peng *et al.*, 1999; Börner *et al.*, 2000), for identifying quantitative trait loci (QTLs) (Parker *et al.*, 1998) and for molecular marker-assisted selection (Korzun *et al.*, 1998, Huang *et al.*, 2000). In species other than wheat, microsatellite markers have also been used to ascertain the genetic purity of seeds (Schuster *et al.*, 2004), as well as a means of protecting intellectual property (Bertini *et al.*, 2006; Schuster *et al.*, 2006). However, in order to use microsatellite markers as cultivar-identification tools for the protection of intellectual-property, for example, it is necessary to know the allelic frequency of informative loci in order to calculate random identity-exclusion probability (Schuster *et al.*, 2006).

In this work, we evaluated genetic diversity within a representative compilation of the germplasm of Brazilian wheat, in order to assess allelic frequencies in an informative set of microsatellite loci, thereby permitting a more accurate identification of varieties of Brazilian wheat. Thirty-six cultivars, developed at various research centers, both private and public, were used (Table 1).

DNA samples were extracted from a thirty-seed bulk of each cultivar, obtained from the Coodetec germplasm collection. DNA extraction from the seeds was undertaken according to the protocol described by McDonald *et al.* (1994), but with certain adjustments. Thirty seeds from each cultivar were ground utilizing a MA 048 mill (Marconi), and 50 mg sub-samples of flour collected therefrom. DNA concentration was determined spectrophotometrically (Sambrook *et al.*, 1989).

Microsatellite-loci amplification was performed by using DNA samples in a 20 μL volume containing 12.5 mM Tris-HCl (pH 8.3), 62.5 mM KCl, 2.5 mM MgCl_2_, 125 μM of each of the deoxynucleotides (dATP, dTTP, dGTP e dCTP), 0.4 μM of each primer, one unit of Taq DNA polymerase enzyme and 75 ng of DNA. Amplification was achieved with a 7-min initial step at 94 °C followed by thirty cycles of 1 min at 94 °C, 1 min at 55 °C, 2 min at 72 °C and finally a 7-min step at 72 °C after the 30^th^ cycle. Amplified fragments were separated and revealed on 7% polyacrylamide denaturing gels stained with silver-nitrate.

The polymorphic information content (PIC) of each microsatellite locus was evaluated through allelic frequency:



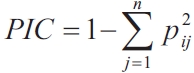


where *p*_ij_ represents the frequency from the *j*^th^ allele to the *i*^th^ primer (Anderson *et al.*, 1993).

Genetic relationships among accessions were evaluated through a dissimilarity matrix, using the complement of a similarity index for co-dominant and multi-allelic data with the aid of GENES software (Cruz, 2006).

From dissimilarity assessments, cultivars were clustered by means of the Tocher optimization method, the hierarchical unweighted pair-group method with arithmetic means analysis (UPGMA), Ward's method and complete and single linkage methods. A cophenetic correlation analysis between the original dissimilarity matrix and the dissimilarity matrices obtained from dendograms was carried out to define the hierarchical clustering method which best represented the original data. Clustering analysis using hierarchical methods was undertaken with STATISTICA software (StatSoft Inc Tulsa, OK, USA) and Tocher optimization clustering analysis with GENES software (Cruz, 2006).

Besides eight unmapped loci (Table 2), 12 genome-A loci, 13 genome-B loci and 10 genome-D loci were evaluated. From these 43 loci, 23 presented polymorphisms among the 36 wheat cultivars analyzed (54%). In these 23 polymorphic loci, the numbers of alleles observed per locus ranged from two to five, making a total of 74 alleles, with a mean of 3.2 alleles per locus. PIC values calculated in order to estimate the informativeness of each polymorphic locus, varied from 0.20 to 0.79, with a mean of 0.49 (Table 2).

The variabilities obtained in A, B and D genomes were similar. In genome A, six out of the twelve loci evaluated were polymorphic (50%), with 21 alleles (3.5 alleles per locus), whereas, in genome B, seven out of thirteen loci were polymorphic (54%), with 18 alleles (2.6 alleles per locus), and in genome D, six out of ten SSR loci were polymorphic (60%), with s17 alleles (2.8 alleles per locus).

Among the eight unmapped SSR loci, four were polymorphic (50%), with 18 alleles (4.5 alleles per locus). PIC values were 0.54; 0.49; 0.38 and 0.60 for A, B and D genomes, as well as the unmapped loci, respectively.

Genetic distances among the cultivars ranged from 0.10, between Ocepar 18 and BRS 207, to 0.88 between CD 101 and Fundacep 46, with a mean distance of 0.48. The highest frequencies of genetic distance occurring between 0.40 and 0.60 (Figure S1, in supplementary material).

Table 1 sets forth the genealogy of the 36 cultivars. A group of three cultivars (Fundacep 46, 50 and 52) and another pair of cultivars (BRS208 and 210) could not be distinguished by genealogy. There were 74 different parents in the genealogy of these wheat cultivars, of which a few participated in more than one genealogy. From the 630 parentage coefficients (CP) possible between these 36 cultivars, only 24 were different from zero. Therefore, it was not possible to correctly group these cultivars by genealogy alone.

By using molecular markers, it was possible to distinguish all thirty-six cultivars and group these by genetic similarity. We used four hierarchical methods. Cophenetic correlations obtained between the original dissimilarity matrix and matrices from dendograms were 67%, 42%, 46% and 53% for the UPGMA, Ward's, complete-linkage and single-linkage methods, respectively. Due to its higher degree of cophenetic correlation, the UPGMA approach (Figure 1) was chosen for graphical representation of cultivar clusters.

**Figure 1:**
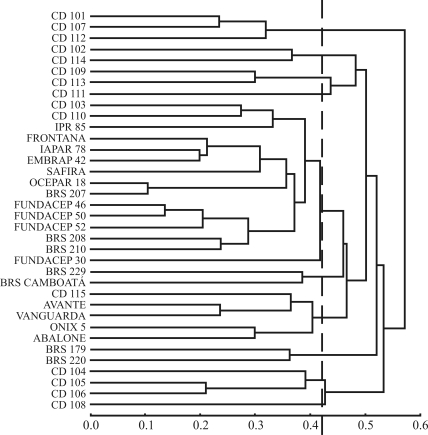
Clustering assessment obtained by UPGMA based on dissimilarity measurements of thirty-six Brazilian wheat cultivars. The hatched line indicates the cutoff for cluster formation.

Furthermore, clusters obtained by UPGMA, when a cutoff is at a distance of 0.42, paralleled those obtained by the Tocher optimization method (Table 3). Only two of the 36 cultivars presented some clustering divergence between methods. Cultivar CD 110 was clustered in group 1 by UPGMA and in group 7 by Tocher. Cultivar CD 104, clustered in group 2 by UPGMA, remained isolated in group 12 by Tocher. The other 34 cultivars were clustered similarly by both methods, thus indicating a high degree of clustering consistency.

The 13 clusters formed revealed high genetic diversity among the wheat cultivars. Group 1, consisting of 13 cultivars by the Tocher method and 14 by the UPGMA, contained cultivars from all origins. The three Fundacep cultivars with the same genealogy (Fundacep 46, 50 and 52) were closely clustered. The two cultivars from Embrapa, with the same genealogy (BRS208 and 210), were also clustered within this group (Figure 1). Of the 18 Coodetec germplasm cultivars, 14 were clustered either in groups containing exclusively Coodetec cultivars or were not clustered together with any other cultivar (groups 2, 3, 5, 7, 9, 12 and 13) at all. Even so, there was high genetic variability among these cultivars themselves, seeing that seven different groups were obtained.

Similarly, there was a tendency for OR cultivars to form unique groups. Group 6 contained OR seeds exclusively, and group 4 contained only one cultivar from another origin. Of the eight Embrapa cultivars evaluated, four were clustered in group 1 along with others, the remaining four forming two exclusive groups, 8 and 10.

Table 2 shows the frequencies of all alleles revealed in polyacrylamide gels. Since fragment size characterization is not completely reproducible in this system, a representative cultivar was placed for each allele, so as to enable recognizing alleles identical to those observed in this work when using these same cultivars in future studies. This is important, especially in cases where it is necessary to define the genetic identity of a certain cultivar. On knowing allele frequency, identity and exclusion probabilities can then be calculated (Schuster *et al.*, 2006).

Results obtained with polymorphic primers in this work were consistent with those reported by other authors. Akkaya and Buyukunal-Bal (2004) obtained PIC values ranging from 0.36 to 0.87, with a mean of 0.68, when evaluating 19 SSR highly polymorphic loci in 11 wheat cultivars. Ahmed (2002) observed from two to eight alleles per locus, with a mean of 3.6, in 13 wheat genotypes of diverse origin analyzed with 43 SSR markers. We observed a mean of 3.2 alleles per locus within a range from two to five, and for PIC values ranging from 0.20 to 0.79, a mean of 0.49.

The microsatellite profiles, characterized with 23 pairs of polymorphic markers, showed that the loci employed delineated all the 36 cultivars. Thus, these markers can be used to define the genetic profile of each cultivar. These genetic profiles, or fingerprints, may be useful in the protection of cultivars, for ensuring genetic purity and for generating further information to underpin breeding programs (Bertini *et al.*, 2006). In addition and due to the allelic frequencies obtained in each locus, it is possible to acquire genetic identity and exclusion probabilities in cases involving the protection of intellectual-property (Schuster *et al.*, 2006).

To date, no evaluation of Brazilian wheat germplasm by using microsatellite markers has been undertaken. The markers applied here were found to be polymorphic, with a potential for germplasm characterization. With this in mind, these should be chosen according to their informativeness and measured by PIC magnitude values.

The results obtained in this study revealed that there is significant genetic divergence among Brazilian wheat cultivars, so that breeding programs can exploit expressive genetic variability by using only adapted germplasm. This observation can be set forth based on molecular data as much as genealogy. However, genealogy cannot group cultivars by genetic similarity, whereas molecular data can do so. Also, our results revealed that cultivars may be grouped according to origin.

On evaluating 23 Brazilian wheat cultivars, Bertan *et al.* (2006) arrived at similar results. In order to separate aluminum-susceptible and -resistant cultivars, these authors obtained four large groups of cultivars, most of which were delineated according to the supplier. Vieira *et al.* (2007), on using AFLP markers to evaluate genetic diversity in another set of 19 Brazilian wheat cultivars, found that out of 11 cultivars from EMBRAPA, seven did not group closely with any cultivar in the same group, independent of the origin.

Results obtained in this work show that a highly variable wheat germplasm has been adapted for use in Brazil. Therefore, it is possible to widely exploit this vast variability in segregating populations within breeding programs, by simply using only adapted cultivars. The results also provide information on the informativeness of those microsatellite loci and allelic frequencies that may be applied in evaluating still further the wheat germplasm used in Brazil, as well as in protecting intellectual property.

## Supplementary Material

The following online material is available for this article:

Figure S1 Frequency distribution of genetic distances.

This material is available as part of the online article from http://www.scielo.br/gmb.

## Figures and Tables

**Table 1:** - Genealogy and suppliers of wheat cultivars used in the genetic diversity study.

Supplier	Nature	Cultivar	Genealogy
EMBRAPA	Public	BRS 179	BR 35/PF 8596/3/ PF 772003*2/PF 813//PF 83899
		BRS 207	SERI 82/PF 813
		BRS 208	CPAC89118/3/BR23//CEP19/PF85490
		BRS 210	CPAC89118/3/BR23//CEP19/PF85490
		BRS 220	EMBRAPA16/TB108
		BRS 229	EMB 27*3//BR 35/BUCK PONCHO
		BRS Camboatá	HULHANEGRA/CNT 7//AMIGO/CNT 7
		Embrapa 42	LAP 689/MS 7936

COODETEC	Cooperative	CD 101	AU/UP301//OCEPAR 12-MAITACA
		CD 102	IAC5/ALDAN"S"//CEP 7780
		CD 103	PG 864/OCEPAR 14
		CD 104	PFAU'S'/IAPAR 17
		CD 105	PFAU”S”/2*OCEPAR 14//IAPAR 41
		CD 106	PG 864/GENARO
		CD 107	COCORAQUE*2/BR 23//BR 35
		CD 108	TAM200/TURACO
		CD 109	MUNIA/BAGULA
		CD 110	ANAHUAC 75/EMBRAPA 27
		CD 111	EMBRAPA 27/OCEPAR 18//ANAHUAC 75
		CD 112	IOC 905/pg 877
		CD 113	EMBRAPA 27/OC 946
		CD 114	PF 89232/OC 938
		CD 115	OC 926//BTU/pg 868
		Ocepar 18	KVZ/BUHO ‘S'//KAL/BB

IAPAR	Public	IPR 85	IAPAR 30/BR18
		Iapar 78	VEE'S/'BOW'S

FUNDACEP	Fundation	Fundacep 30	BR 32/CEP 21//CNO 79
		Fundacep 46	CEP 88132/pg 876/3/BR 34//CRDN
		Funadcep 50	CEP 88132/pg 876/3/BR 34//CRDN
		Fundacep 52	CEP 88132/pg 876/3/BR 34//CRDN

OR Sementes	Private	Avante	PF89232/2* OR1
		Abalone	ORL92299/3/ORL92171//EMB16/OR1/4/RUBI
		Ônix 5	CEP 24/RUBI'S'
		Vanguarda	OR 1/ORL92177//EMB16/OR1
		Safira	PF 9099/OR 1//GRANITO

Public domain	-	Frontana	FRONTEIRA/MENTANA

**Table 2:** - Microsatellite markers used in assessment of genetic diversity of Brazilian wheat cultivars.

Locus	Chromosome location (cM - GL)^#^	Number of alleles	Frequency of alleles	Representative alleles^§^	PIC
Xgwm 136	3.9-1A	5	0.12; 0.31; 0.26; 0,24; 0.07	CD 115; CD 114; FRONTANA; CD 112; ABALONE	0.76
Xgwm 164	40.5-1A	4	0.22; 0.17; 0.55; 0.06	CD 112; CD 105; CD 104; CD 111	0.61
Xgwm 135	55.2-1A	3	0.18; 0.29; 0.53	CD 113; CD 102; CD 104	0.60
Xgwm 403	64.4-1B	1	1.00	All	-
Xgwm 140	102.1-1B	1	1.00	All	-
Xgwm 337	39.5-1D	4	0.06; 0.10; 0.61; 0.23	BRS 208; CD 114; CD 105; CD 104	0.56
Xgwm 232	130.4-1D	2	0.11; 0.89	CD 102; AVANTE	0.20
Xgwm 359	54.4-2A	1	1.00	All	-
Xgwm 265	112.3-2A	1	1.00	All	-
Xgwm 257	12.3-2B	2	0.20; 0.80	CD 112; CD 104	0.32
Xgwm 261	? - 2D	2	0.39; 0.61	CD 104; IPR 85	0.48
Xgwm 102	36.0-2D	3	0.03; 0.10; 0.87	CD 114; CD 105; CD 103	0.23
Xgwm 301	78.8-2D	1	1.00	All	-
Xgwm 369	18.8-3A	1	1.00	All	-
Xgwm 32	49.5-3A	3	0.11; 0.86; 0.03	CD 111; CD 104 ; ONIX 5	0.25
Xgwm 285	65.3-3B	2	0.54; 0.46	FRONTANA; CD 104	0.50
Xgwm 108	96.1-3B	3	0.13; 016; 0.71	CD 105; CD 108; CD 103	0.46
Xgwm 299	122.6-3B	1	1.00	All	-
Xgwm 161	15.7-3D	3	0.06; 0.26; 0.68	BRS 179; CD 115; CD 111	0.47
Xgwm 456	52.9-3D	1	1.00	All	-
Xgwm 4	16.1-4A	1	1.00	All	-
Xgwm 160	77.1-4A	3	0.22; 0.57; 0.20	CD 105; BRS 210; CD 115	0.58
Xgwm 165	27.1-4B	2	0.85; 0.15	CD 105; CD 104	0.25
Xgwm 149	30.5-4B	2	0.65; 0.34	CD 104; FUNDACEP 52	0.45
Xgwm 194	95.4-4D	3	0.80; 0.17; 0.03	CD 104; CD 115; CD 102	0.32
Xgwm 234	1.8-5B	1	1.00	All	-
Xgwm 213	32.5-5B	1	1.00	All	-
Xgwm 212	107.0-5D	1	1.00	All	-
Xgwm 459	5.0-6A	1	1.00	All	-
Xgwm 219	62.6-6B	3	0.61; 0.19; 0.20	CD 204; CD 208; CD 205	0.79
Xgwm 233	6.6-7A	3	0.13; 0.71; 0.16	CD 110; CD 104; CD 111	0.46
Xgwm 276	85.6-7A	1	1.00	All	-
Xgwm 43	33.7-7B	1	1.00	All	-
Xgwm 302	57.2-7B	1	1.00	All	-
Xgwm 44	43.9-7D	4	0.16; 0.13; 0.52; 0.16	CD 112; CD 114; BRS 229; ÔNIX 5	0.65
Xgwm 247	*	5	0.03; 0.03; 0.50; 0.22; 0.22	CD 105; CD 108; CD 104; AVANTE; BRS 220	0.65
Xgwm 304	*	4	0.06; 0.06; 0.76; 0.12	FUNDACEP 30; CD 112; BRS 229; CD 104	0.45
Xgwm 332	*	1	1.00	All	-
Xgwm 155	*	4	0.05; 0.39; 0.53; 0.03	FUNDACEP 50; CD 114; CD 104; CD 109	0.57
Xgwm 372	*	5	0.37; 0.12; 0.29; 0.06; 0.16	CD 111; ABALONE; BRS CD 115; CD 105	0.73
Xgwm 126	*	1	1.00	All	-
Xgwm 132	*	1	1.00	All	-
Xgwm 268	*	1	1.00	All	-

#Location of markers in wheat genetic map (Song *et al.*, 2005). *Unmapped loci. ?Unknown position. ^§^Representative cultivars of alleles in each locus. Each cultivar represents the allele, whose frequency is shown in the previous column, in the same order. Primer sequences can be obtained in http://wheat.pw.usda.gov/cgi-bin/graingenes/browse.cgi?class = marker.

**Table 3:** - Grouping of 36 wheat cultivars assessed from genetic distances obtained by microsatellite marker data, using Tocher's and the UPGMA methods.

Group	Tocher	UPGMA
1	BRS 210; BRS 208; FUNDACEP 52; FUNDACEP 50; FUNDACEP 46; OCEPAR18; BRS 207; EMBRAP42; SAFIRA; IAPAR 78; FRONTANA; CD 103; IIPR 85	BRS 210; BRS 208; FUNDACEP 52; FUNDACEP 50; FUNDACEP 46; OCEPAR18; BRS 207; EMBRAP42; SAFIRA; IAPAR 78; FRONTANA; CD 103; IIPR 85; CD 110
2	CD 105; CD 106	CD 105; CD 106; CD 104
3	CD 101; CD 107; CD 112	CD 101; CD 107; CD 112
4	AVANTE; VANGUARDA; CD 115	AVANTE; VANGUARDA; CD 115
5	CD 109; CD 113	CD 109; CD 113
6	ONIX 5; ABALONE	ONIX 5; ABALONE
7	CD 111; CD 110	CD 111
8	BRS 179; BRS 220	BRS 179; BRS 220
9	CD 102; CD 114	CD 102; CD 114
10	BRS 229; BRS CAMBOATÁ	BRS 229; BRS CAMBOATÁ
11	FUNDACEP 30	FUNDACEP 30
12	CD 104	-
13	CD 108	CD 108
