# Supplementary material for: Genetic variability in Brazilian wheat cultivars assessed by microsatellite markers
Source: Genet Mol Biol. 2009 Sep 1;32(3):557–63. doi: 10.1590/S1415-47572009005000045 (PMC3036063; doi:10.1590/S1415-47572009005000045)
Supplement: Figure S1 — Frequency distribution of genetic distances. [file gmb-32-3-557-suppl1.pdf]

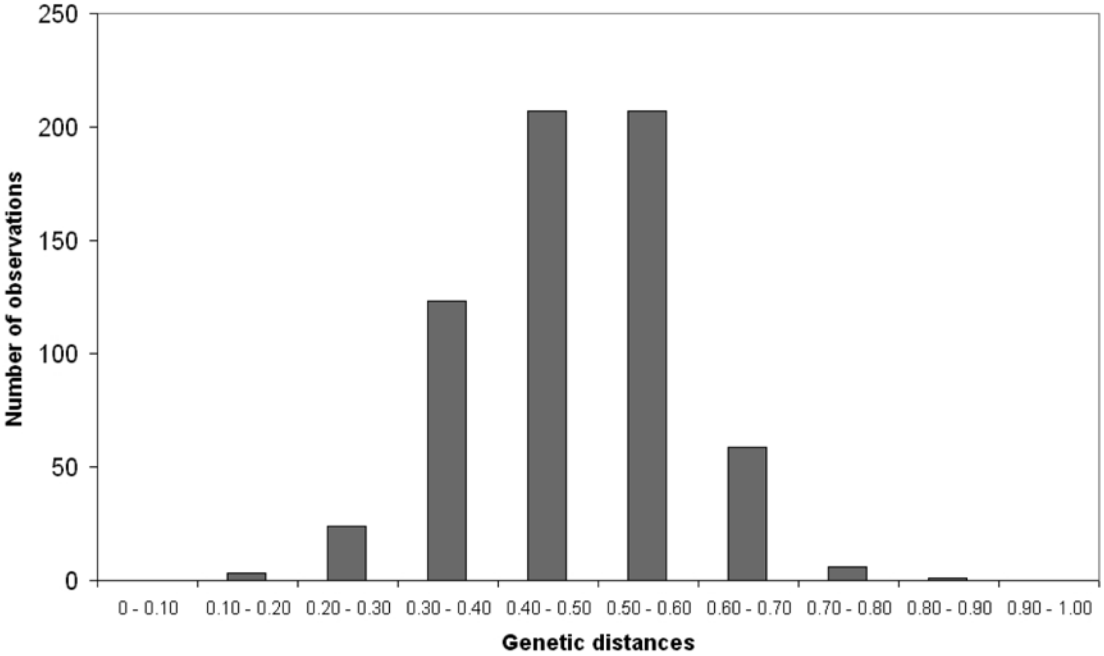

Figure S1 - Frequency distribution of genetic distances obtained in thirty-six wheat cultivars with twenty-three SSR polymorphic loci.
